# Immune Infiltration in Gastric Cancer Microenvironment and Its Clinical Significance

**DOI:** 10.3389/fcell.2021.762029

**Published:** 2022-02-17

**Authors:** An Zhi Zhang, Xin Yuan, Wei Hua Liang, Hai Jun Zhang, Ya Li, Yu Fang Xie, Jiang Fen Li, Chen Hao Jiang, Fan Ping Li, Xi Hua Shen, Li Juan Pang, Hong Zou, Wen Hu Zhou, Feng Li, Jian Ming Hu

**Affiliations:** ^1^ Department of Pathology/NHC Key Laboratory of Prevention and Treatment of Central Asia High Incidence Diseases, The First Affiliated Hospital, School of Medicine, Shihezi University, Shihezi, China; ^2^ Department of Pathology, Jiaxing University Affiliated Women and Children Hospital (Jiaxing Maternity and Child Health Care Hospital), Jiaxing University, Jiaxing, China; ^3^ Xiangya School of Pharmaceutical Sciences, Central South University, Changsha, China; ^4^ Department of Pathology, Beijing Chaoyang Hospital, Capital Medical University, Beijing, China

**Keywords:** GC, TIICs, immune genes, tumor microenvironment, prognosis

## Abstract

Immunotherapy has developed rapidly and has gradually become one of the important methods for treatment of gastric cancer (GC). The research on tumor infiltrating immune cells (TIICs) and immune-related genes in the tumor microenvironment (TME) greatly encourages the development of immunotherapy. The devolution algorithm (CIBERSORT) was applied to infer the proportion of 22 TIICs based on gene expression profiles of GC tissues, which were downloaded from TCGA and GEO. TCGA was utilized to analyze the differential expression of immune-related genes, and explore the potential molecular functions of these genes. We have observed the enrichment of multiple TIICs in microenvironment of GC. Some of these cells were closely related to tumor mutational burden (TMB), microsatellite instability (MSI), Fuhrman grade, and TNM staging. Survival analysis showed that the infiltration level of CD8^+^ T cells, activated CD4^+^ memory T cells and M2 macrophages were significantly related to the prognosis of GC patients. The functional enrichment analysis of immune-related genes revealed that these genes were mainly associated with cytokine activation and response. Four significant modules were screened by PPI network and 20 key genes were screened from the modules. The expression levels of CALCR and PTH1R are strikingly related to the expression of immune checkpoint and the prognosis of GC patients. The type and number of TIICs in microenvironment of GC, as well as immune-related genes are closely related to tumor progression, and can be used as important indicators for patient prognosis assessment.

## Introduction

Gastric cancer (GC) remains an important cancer worldwide and is responsible for over one million new cases in 2020 and an estimated 769,000 deaths, ranking fifth for incidence and fourth for mortality globally (Sung et al., 2021). Although, with the advances in diagnostic and therapeutic techniques, prognosis of GC patients has improved significantly, but the clinical outcome is not satisfactory. GC is a very heterogeneous tumor (Gao et al., 2018). The response of tumors to drugs varies greatly between primary lesions and metastases, or at different stages of treatment. It is difficult to achieve a satisfactory therapeutic effect by a single method. In addition to classic surgery and systemic chemotherapy, immunotherapy has also been proven to have the potential to eradicate cancer (Zacharakis et al., 2018). It can be used as a potential alternative strategy for GC treatment (Beavis et al., 2017). Research on markers of immune efficacy can greatly promote the development of immunotherapy. The existing immune efficacy markers can be roughly divided into two categories: the first category is molecular markers related to tumor neoantigen load, such as microsatellite instability (MSI) or tumor mutational burden (TMB) increase; The second category is related to the tumor microenvironment (TME), including the expression of PD-L1 protein, tumor cell infiltration, and copy number variation (Cristescu et al., 2018). These two types of markers are involved in the different stages of tumor immunotherapy and form a unified whole. The lack of one cannot reflect the full picture of tumor immunotherapy. Combining two or more methods that reflect the immune status of the tumor microenvironment to form a composite biomarker for efficacy prediction is a more effective and universal method for predicting the efficacy of immunotherapy. However, the immunotherapy of GC is still at an early stage, and more research is needed to provide a theoretical basis for its development.

The tumor microenvironment (TME) is a complex system composed of extracellular matrix, chemokines, cytokines, and non-tumor cells (Yang et al., 2018). Tumor infiltrating immune cells (TIICs), mainly B cells, T cells and tumor-associated macrophages, are an important part of non-tumor cells. Some studies have further reported that TIICs, as independent prognostic factors of various cancers, play a key role in promoting, and inhibiting cancer growth (Lee et al., 2014; Liu et al., 2018; Nishino et al., 2017; Turley et al., 2015; Xiong et al., 2018). Genome analysis has become one of the principal methods for discovering new biological targets of GC in the world (Atlas 2014; Oh et al., 2018). Certain genomic features, such as MSI, TMB, and tumor aneuploidy have been associated with tumor immunity and im-munotherapy response and may serve as predictive biomarkers for cancer immunotherapy (Yin et al., 2019). Recently, TMB and MSI has been shown to be associated with clinical outcomes in diverse cancers, such as melanoma (Hugo et al., 2016), non-small-cell lung cancer (Hellmann et al., 2018; Xu et al., 2000), and colorectal cancer (Fabrizio et al., 2018; Goodman et al., 2019). In addition, some studies have revealed the importance of tumor-related structures and up-regulated signaling pathways in TME (Su et al., 2018). It is found that immune-related genes are closely linked to TIICs and play an important role in TME (Brouwer-Visser et al., 2018; Omrane and Benammar-Elgaaied 2015; Qu et al., 2020). Immune checkpoint inhibitors, such as CTLA-4 and PD-1/PDL1, has received widespread attention in recent years (Sharma and Allison 2015a; Sharma and Allison 2015b). In the currently discovered immune checkpoint, the interaction between programmed death receptor 1 (PD-1) and its ligand programmed death ligand 1 (PD-L1) has been identified as an important immunosuppressive mechanism in cancer (Dong et al., 2002; Freeman et al., 2000). However, the interplay between these two distinct categories of biomarkers has not been well characterized across gastric cancer with respect to their ability either to independently or jointly predict response to immunotherapy or to reveal underlying genomic and/or transcriptomic features of tumor antigenicity and TME. In this study, we used the CIBERSORT algorithm, combined with Gene Expression Omnibus (GEO) and The Cancer Genome Atlas (TCGA) to analyze the infiltration of 22 TIICs in GC. We systematically studied the relationship between TIICs and their relationship with TMB, MSI, clinicopathological characteristics and survival, and deeply explored the possible role of GC immune-related genes in TME and their relationship with immune checkpoints and prognosis. A comprehensive description of the immune components in TME may help explain the response of GC to immunotherapy. At the same time, it also serves to reveal new therapeutic targets and provide new strategies for cancer treatment.

## Materials and Methods

### Gene Expression Datasets

This study made use of data from public datasets. The gene expression profiles were obtained from GEO and TCGA (downloaded in January 2020). Then subjected to background correction and normalization with Perl 5.0 (http://www.perl.org/). Among them, the GSE66229 dataset contains 300 cases of GC tissues and 100 cases of normal tissues. There are 373 cases of downloaded gene transcript data in TCGA, including 343 cases of gastric adenocarcinoma tissues, and 30 cases of normal tissues. Meanwhile, we also collected the clinical characteristics of related cases in TCGA. Subsequently, we organized sample and corresponding clinical data for further analysis. Moreover, we picked out tumor tissues and normal tissues to screen differentially TIICs and immune-related genes to investigate whether there is a difference in TIICs and immune-related genes between different tissues.

### Estimation of TIICs Type Fractions

CIBERSORT is a robust analytic tool that uses gene expression signatures consisting of 547 genes. It characterizes each TIICs subtype and accurately quantifies distinct TIICs compositions using a deconvolution algorithm (Newman et al., 2015; Pan et al., 2020). We upload the data to the CIBERSORT web portal (http://cibersort.stanford.edu) with a number of permutations being set to 100. Cases with CIBERSORT *p* < 0.05, which reflected that the deconvolution results were accurate, would be selected for further analysis. In the present study, a total of 636 samples (100 normal samples and 300 GC samples in GEO; 15 normal samples and 221 GC samples in TCGA) were filtered out. Correlations between different TIICs subtypes were established using the Pearson correlation coefficient.

### Analysis of Differentially Expressed Immune-Related Genes

The somatic mutation data downloaded from the TCGA GDC data portal is used to compare the correlation between different immune cells and TMB. The MSI score of the TCGA sample is based on previous research (Bonneville et al., 2017). Spearman’s coefficient was used to calculate the correlation between immune cell infiltration and TMB and MSI. The result is displayed in the form of a radar curve graph using the R-package “fmsb”.

The differential expression analyses were conducted between GC tissues and normal tissues using the “DEseq2” R package, with parameters of log2 |fold change| > 1 and adj. *p* < 0.05. Then, the obtained differential genes and the immune genes obtained in the Immport database (https://www.immport.org/home) are crossed to obtain immune-related differential genes. Functional enrichment analysis was conducted by using the DAVID website (https://david.ncifcrf.gov) to determine potential functions and pathways. Functional enrichment was conducted for the Gene Ontology (GO) terms including the cellular component, biological process, and molecular function categories, as well as the Kyoto Encyclopedia of Genes, and Genomes (KEGG) pathways. A false discovery rate< 0.05 was used as the cut-off. All immune-related differential genes were inputted into STRING (https://string-db.org) to predict protein-protein interactions. Data of the PPI network were processed by Cytoscape and key genes in significant modules (MCODE score >10 and number of nodes >20) were extracted from the PPI network were used for further analysis. Spearman’s rank test was used to analyze the correlation between immune-related genes and immune checkpoints (such as PD-1, PD-L1, CTLA4, LAG3, and VSTM3). Meanwhile, use the DAVID website to perform functional enrichment analysis on important modules, and enter key genes into TIMER (https://cistrome.shinyapps.io/timer/) to determine the expression of key genes in each tumor, so as to have a deeper understanding of genes.

### Statistical Analysis

Kaplan–Meier analysis and a log-rank test were performed to construct a survival curve and analyze the association of the proportions of 22 TIICs with overall survival. All analyses were conducted by R version 3.6.3 and all statistical tests performed were two-sided. A *p*-value < 0.05 was considered as statistically significant.

## Results

### TIICs in the Microenvironment of GC

**FIGURE 1:**
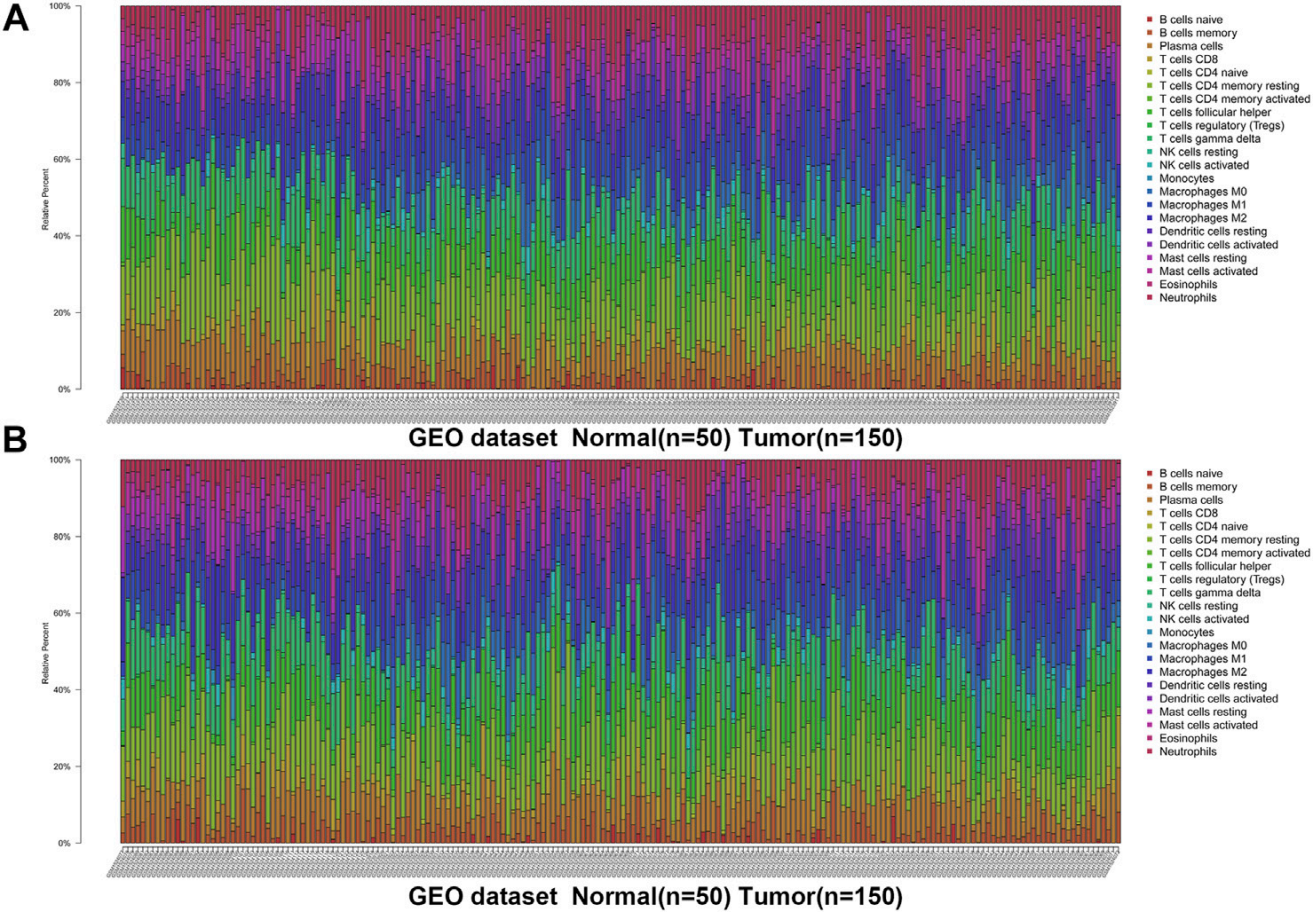
The landscape of immune infiltration in GC and difference of immune infiltration between normal tissues and tumor tissues in GSE66229 dataset. **(A)** Normal tissues (*n* = 50) and GC tissues (*n* = 150). **(B)** Normal tissues (*n* = 50) and GC tissues (*n* = 150).

**FIGURE 2:**
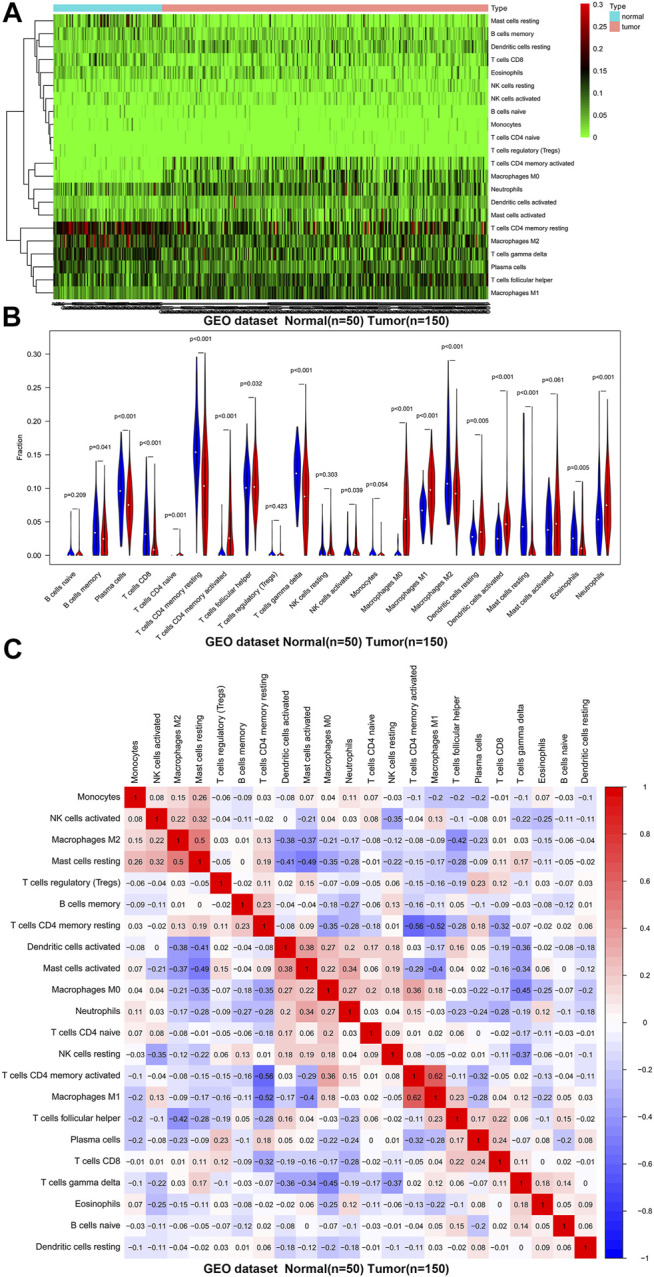
The distribution and correlation analysis of 22 TIICs in GC tissues and normal tissues in GSE66229 dataset. **(A)** Heat map of the 22 immune cell proportions. Green represents low infiltration, black represents medium infiltration and red represents high infiltration. **(B)** Violin plot shows the difference in the proportion of immune cell infiltration. Blue is normal tissues and red is tumor tissues. **(C)** The correlation heat map describes the correlation between TIICs. Red representing positive correlation and blue representing negative correlation.

**FIGURE 3:**
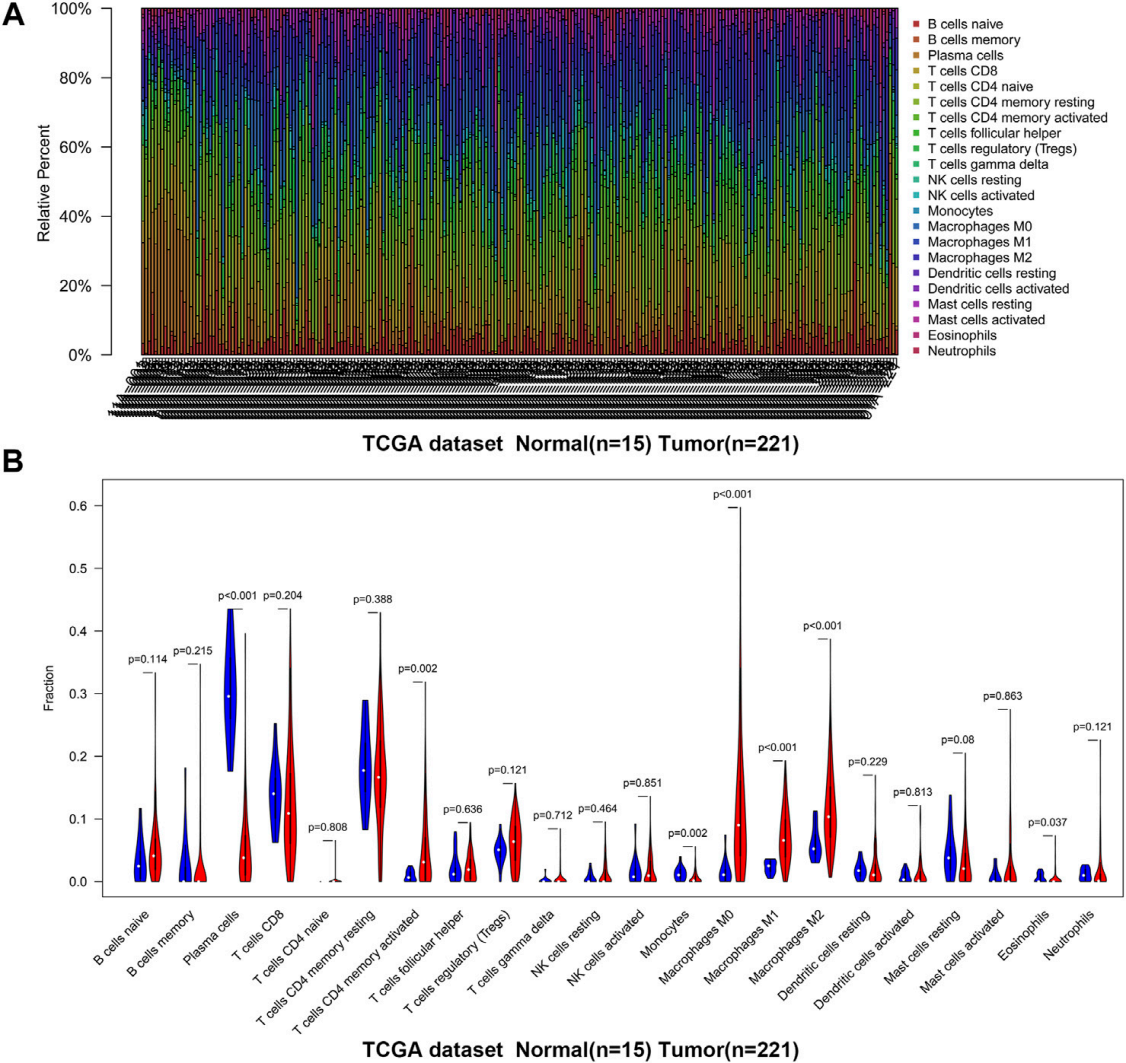
The landscape of immune infiltration in GC and difference of immune infiltration between normal tissues and tumor tissues in TCGA. **(A)** Normal tissues (*n* = 15) and GC tissues (*n* = 221). **(B)** Violin plot shows the difference in the proportion of immune cell infiltration. Blue is normal tissues and red is tumor tissues.

**FIGURE 4:**
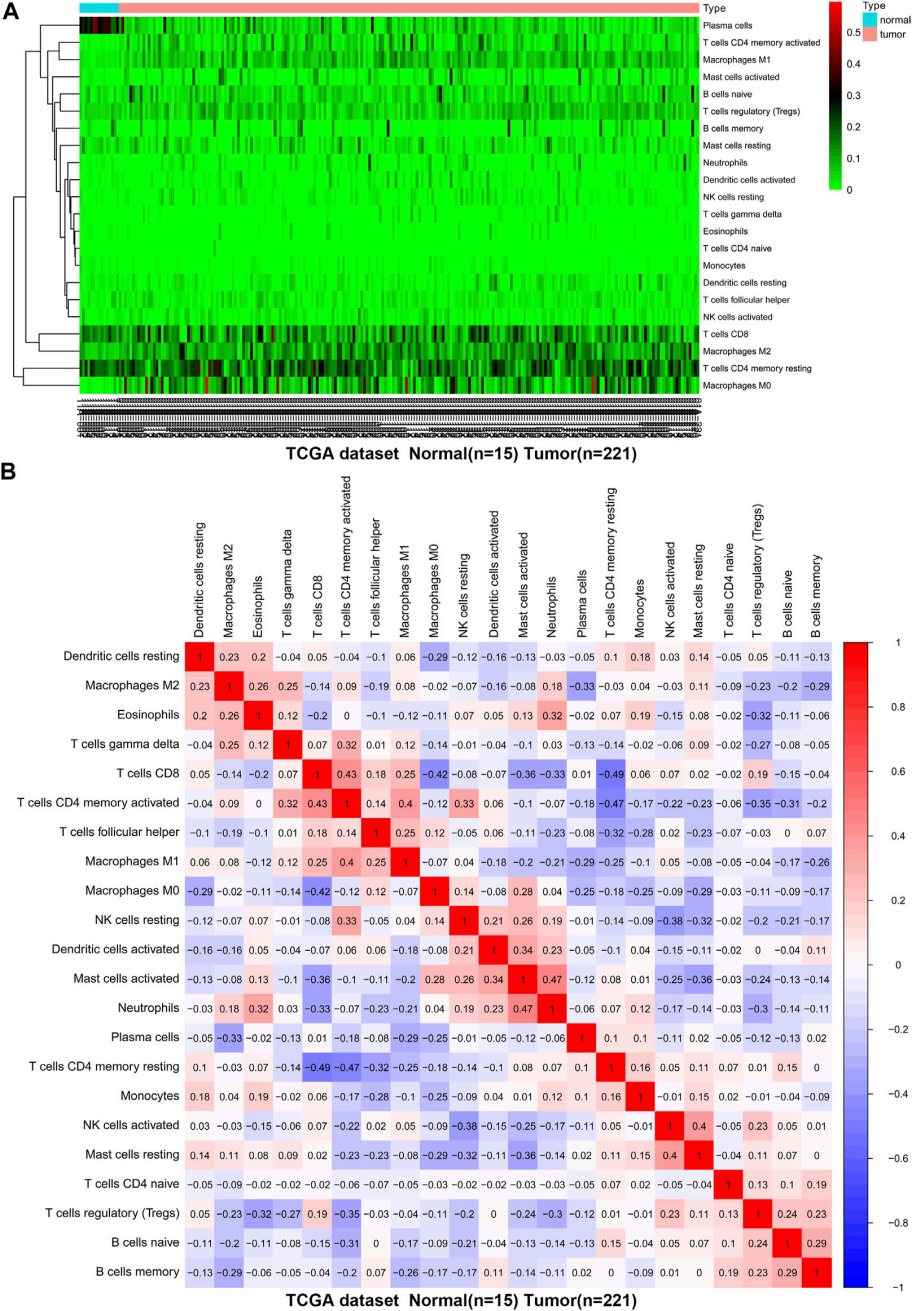
The difference and correlation analysis of immune cell infiltration between GC tissues and normal tissues in TCGA. **(A)** Heat map of the 22 immune cell proportions. Green represents low infiltration, black represents medium infiltration and red represents high infiltration. **(B)** The correlation heat map describes the correlation between TIICs. Red representing positive correlation and blue representing negative correlation.

We first revealed the infiltration of 22 TIICs in GC, and subsequently we investigated the difference between tumor tissues and normal tissues using the CIBERSORT algorithm. Table 1 lists the detailed results. It can be seen from the heat map and histogram that the proportion of TIICs in GEO is significantly different between GC tissues and normal tissues (Figure 1; Figure 2A). Compared with normal tissues, GC tissues contains more activated CD4^+^ T cells, activated CD4^+^ memory T cells, follicular helper T cells, activated NK cells, M0 macrophages, M1 macrophages, resting dendrites cells, activated dendritic cells, and neutrophils (Figure 2B). However, memory B cells, plasma cells, CD8^+^ T cells, resting CD4^+^ memory T cells, γδ T cells, M2 macrophages, resting mast cells, and eosinophils account for a relatively low proportion in GC tissues. The proportions of 22 TIICs were weakly-to-strongly correlated in tumor (Figure 2C). Activated CD4^+^ memory T cells and M1 macrophages showed the strongest positive correlation (Pearson correlation = 0.62), while resting CD4^+^ memory T cells and activated CD4^+^ memory T cells showed the strongest negative correlation (Pearson correlation = 0.56). In addition, resting CD4^+^ memory T cells also showed a moderately negative correlation with M1 macrophages (Pearson correlation = 0.52). Then using TCGA for verification, we found that plasma cells, activated CD4^+^ memory T cells, M0 macrophages, M1 macrophages, resting mast cells and eosinophils have the same trend. The expression of M2 macrophages in tumor tissues was significantly higher than that in normal tissues (Figure 3; Figure 4A). The results of correlation analysis show that neutrophils and activated mast cells have the strongest positive correlation (Pearson correlation = 0.47). Resting CD4^+^ memory T cells and CD8^+^ T cells showed the strongest negative correlation (Pearson correlation = 0.49, Figure 4B). Altogether, these results revealed that the immune response of GC acted as an intricate network and proceeded in a tightly regulated way.

**TABLE 1:** Comparison of 22 TIICs proportion between GC and normal tissues in GEO and TCGA.

Cell type	GEO			TCGA		
	Normal tissue	Tumor tissue	P	Normal tissue	Tumor tissue	P
B cells naive	0.0036 ± 0.0115	0.0028 ± 0.0099	0.209	0.0334 ± 0.0333	0.0510 ± 0.0507	0.114
B cell memory	0.0267 ± 0.0308	0.0290 ± 0.0258	0.041	0.0259 ± 0.0488	0.0182 ± 0.0522	0.215
Plasma cells	0.0936 ± 0.0380	0.0760 ± 0.0324	<0.001	0.3050 ± 0.0751	0.0524 ± 0.0581	<0.001
T cells CD8	0.0374 ± 0.0360	0.0213 ± 0.0282	<0.001	0.1396 ± 0.0494	0.1249 ± 0.0898	0.204
T cells CD4 naive	0.0000 ± 0.0000	0.0014 ± 0.0053	0.001	0.0000 ± 0.0000	0.0002 ± 0.0043	0.808
T cells CD4 memory resting	0.1571 ± 0.0537	0.1035 ± 0.0643	<0.001	0.18445 ± 0.0600	0.1679 ± 0.0841	0.388
T cells CD4 memory resting	0.1571 ± 0.0537	0.1035 ± 0.0643	<0.001	0.1845 ± 0.0600	0.1679 ± 0.0841	0.388
T cells CD4 memory activated	0.0045 ± 0.0117	0.0401 ± 0.0443	<0.001	0.0083 ± 0.0084	0.0483 ± 0.0544	0.002
T cells follicular helper	0.0894 ± 0.0447	0.1039 ± 0.0388	0.032	0.0218 ± 0.0233	0.0232 ± 0.0217	0.636
T cells regulatory (Tregs)	0.0017 ± 0.0070	0.0012 ± 0.0053	0.423	0.0471 ± 0.0194	0.0612 ± 0.0365	0.121
T cells gamma delta	0.1198 ± 0.0343	0.0858 ± 0.0494	<0.001	0.0016 ± 0.0049	0.0042 ± 0.0130	0.712
NK cells resting	0.0103 ± 0.0161	0.0120 ± 0.0179	0.303	0.0056 ± 0.0087	0.0121 ± 0.0198	0.464
NK cells activated	0.0095 ± 0.0171	0.0113 ± 0.0162	0.039	0.0163 ± 0.0227	0.0186 ± 0.0234	0.002
Monocytes	0.0035 ± 0.0113	0.0015 ± 0.0060	0.054	0.0127 ± 0.0102	0.0059 ± 0.0091	0.002
Macrophages M0	0.0038 ± 0.0081	0.0611 ± 0.0458	<0.001	0.0152 ± 0.0188	0.1185 ± 0.1111	<0.001
Macrophages M1	0.0644 ± 0.0213	0.0978 ± 0.0361	<0.001	0.0229 ± 0.0098	0.0719 ± 0.0428	<0.001
Macrophages M2	0.1264 ± 0.0549	0.0928 ± 0.0415	<0.001	0.0619 ± 0.0253	0.1152 ± 0.0626	<0.001
Dendritic cells resting	0.0279 ± 0.0168	0.0405 ± 0.0323	0.005	0.0190 ± 0.0130	0.0201 ± 0.0274	0.229
Dendritic cells activated	0.0257 ± 0.0181	0.0483 ± 0.0271	<0.001	0.0081 ± 0.0089	0.0126 ± 0.0230	0.813
Mast cells resting	0.0584 ± 0.0563	0.0143 ± 0.0266	<0.001	0.0450 ± 0.0372	0.0318 ± 0.0387	0.08
Mast cells activated	0.0405 ± 0.0308	0.0535 ± 0.0464	0.061	0.008 ± 0.0123	0.0228 ± 0.0466	0.863
Eosinophils	0.0271 ± 0.0230	0.0225 ± 0.0280	0.005	0.0056 ± 0.0072	0.0038 ± 0.0114	0.037
Neutrophils	0.0576 ± 0.0419	0.0783 ± 0.0435	<0.001	0.0110 ± 0.0091	0.0140 ± 0.0304	0.121

Significance of bold values are p <0.05.

### The Relationship Between TIICs and TMB and MSI in the Microenvironment of GC

**FIGURE 5:**
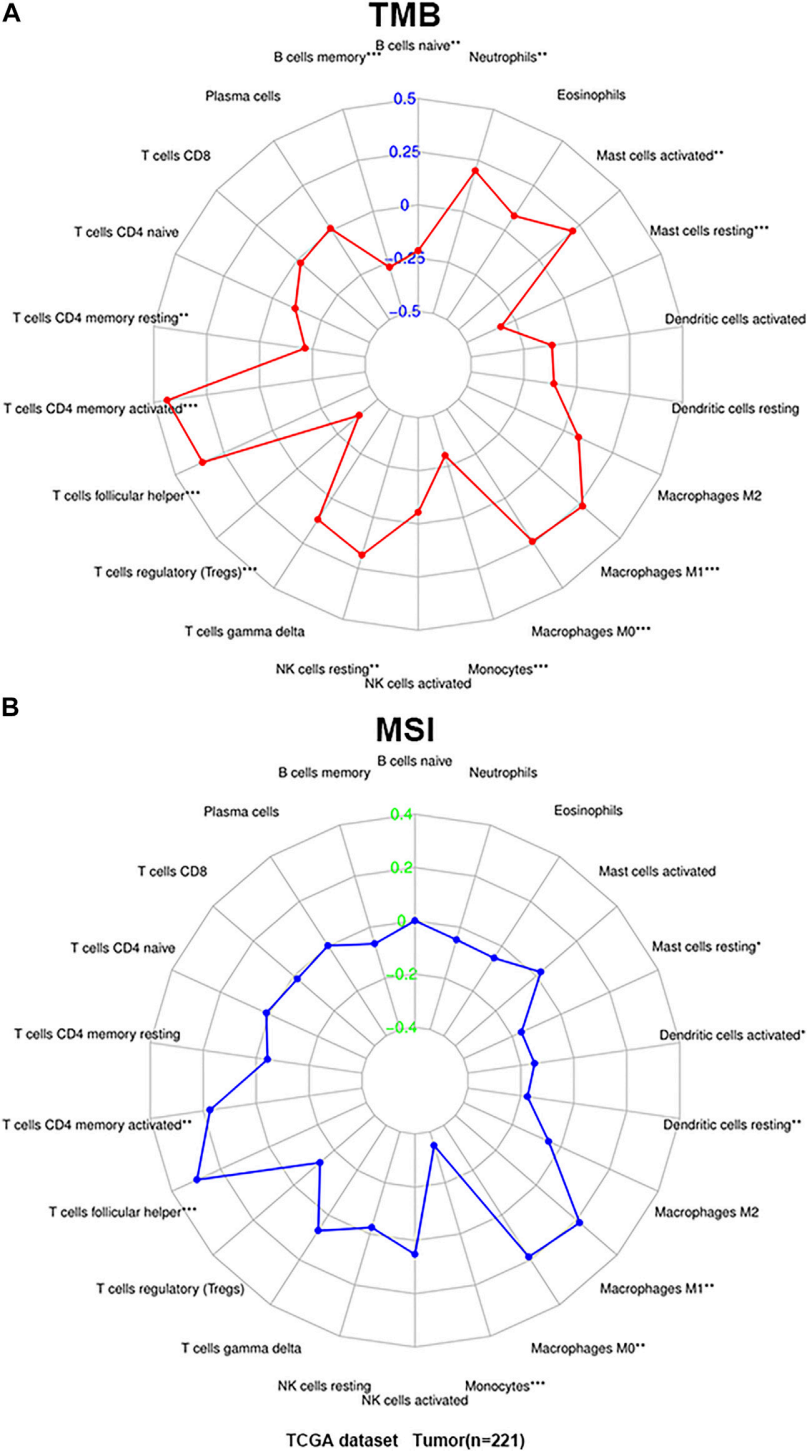
The correlation of TIICs with microsatellite instability (MSI) and tumor mutational burden (TMB). **(A)** Radar map of correlation between TIICs and TMB. **(B)** Radar map of correlation between TIICs and MSI. Spearman’s coefficient was used to calculate the correlation between immune cell infiltration and TMB and MSI. *p < 0.05, **p < 0.01, and ***p < 0.001.

Then we analyzed the correlation between TIICs in the microenvironment and TMB and MSI to determine whether there is an interaction between TIICs and TMB and MSI. The results demonstrated that activated CD4^+^ memory T cells (*p* < 0.001), follicular helper T cells (*p* < 0.001), resting NK cells (*p* < 0.01), M0 macrophages (*p* < 0.001), M1 macrophages (*p* < 0.001), activated mast cells (*p* < 0.01) and neutrophils (*p* < 0.01) were all positively correlated with TMB, while a negative correlation between naive B cells (*p* < 0.01), memory B cells (*p* < 0.001), resting CD4^+^ memory T cells (*p* < 0.01), T cells regulatory (Tregs) (*p* < 0.001), monocytes (*p* < 0.001) and resting mast cells (*p* < 0.001), and TMB (Figure 5A). In addition, the infiltration of activated CD4^+^ memory T cells (*p* < 0.01), follicular helper T cells (*p* < 0.001), M0 macrophages (*p* < 0.01), and M1 macrophages (*p* < 0.01) was significantly associated with increased MSI, while monocytes (*p* < 0.001), resting dendritic cells (*p* < 0.01), activated dendritic cells (*p* < 0.05), and resting mast cells (*p* < 0.05) was negatively associated with MSI (Figure 5B). In summary, TIICs in the microenvironment of GC are closely related to TMB and MSI.

### The Role of TIICs in the Progression and Prognosis of GC

**FIGURE 6:**
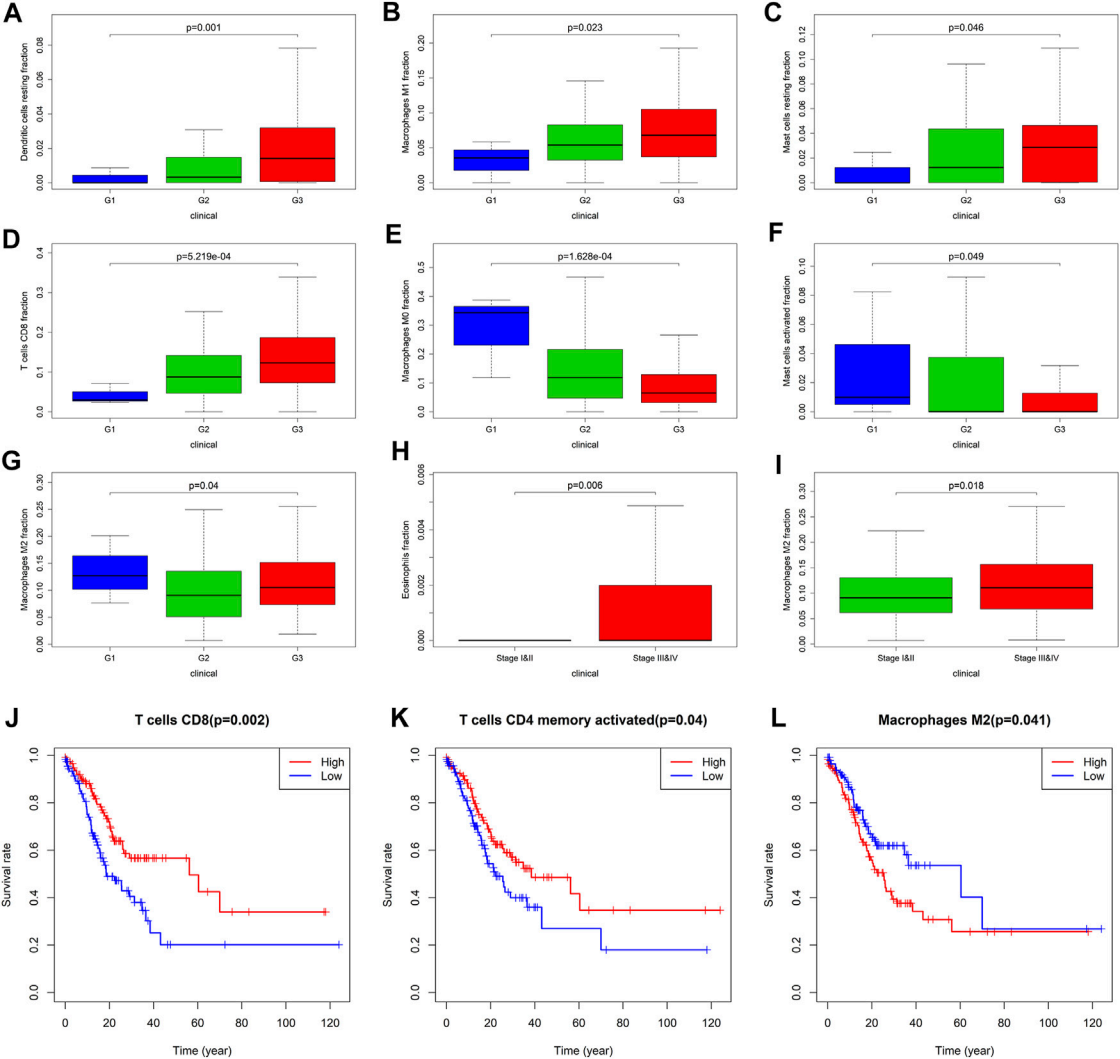
Correlation analysis between TIICs and clinical characteristics and prognosis of patients with GC. **(A–G)** are respectively resting dendritic cells, M1 Macrophages, resting mast cells, CD8^+^ T cells, M0 macrophages, activated mast cells, M2 macrophages and the correlation analysis of Fuhrman grade. G1 represents high differentiation, G2 represents moderate differentiation and G3 represents poor differentiation. **(H,I)** are the correlation analysis between eosinophils and M2 macrophages with TNM staging, respectively. **(J)** CD8^+^ T cells, and **(K)** activated CD4^+^ memory T cells is closely related to the better prognosis of the patient. **(L)** M2 macrophages is closely related to the poor prognosis of patients.

Owning to the missing survival data in GEO, we investigated whether there was statistical significance between immune cells in TCGA, and whether it was statistically relationship with GC patients’ overall survival and other clinical characteristics. Through analysis, it revealed for the first time the relationship between different TIICs and Fuhrman grade or TNM staging of GC (Figures 6A–I, Supplementary Figure S1). The results showed that the proportion of CD8^+^ T cells (*p* < 0.001), resting dendritic cells (*p* = 0.001), M1 macrophages (*p* = 0.023), and resting mast cells (*p* = 0.046) increased with the increase of Fuhrman grade. The ratio of M0 macrophages (*p* < 0.001) and activated mast cells (*p* = 0.049) decreased with the increase of Fuhrman grade. Interestingly, the proportion of M2 macrophages (*p* = 0.04) was the lowest at G2 level, and showed a trend of decreasing first and then increasing with the increase of Fuhrman grade. TIICs were significantly related to TNM staging are eosinophils (*p* = 0.006) and M2 macrophages (*p* = 0.018), and the proportion of these two kinds of TIICs increased with the increase of TNM staging. After limiting CIBERSORT screening to *p* < 0.05, 237 patients can obtain overall survival data. The corresponding survival curve was shown in Figures 6J–L; Supplementary Figure S2. We found that CD8^+^ T cells (*p* = 0.002), activated CD4^+^ memory T cells (*p* = 0.04), and M2 macrophages (*p* = 0.041) were independent prognostic factors for GC patients. CD8^+^ T cells and activated CD4^+^ memory T cells are associated with a better prognosis, while M2 macrophages are associated with a poor prognosis.

### Functional Enrichment Analysis of Immune-Related Differential Genes

**FIGURE 7:**
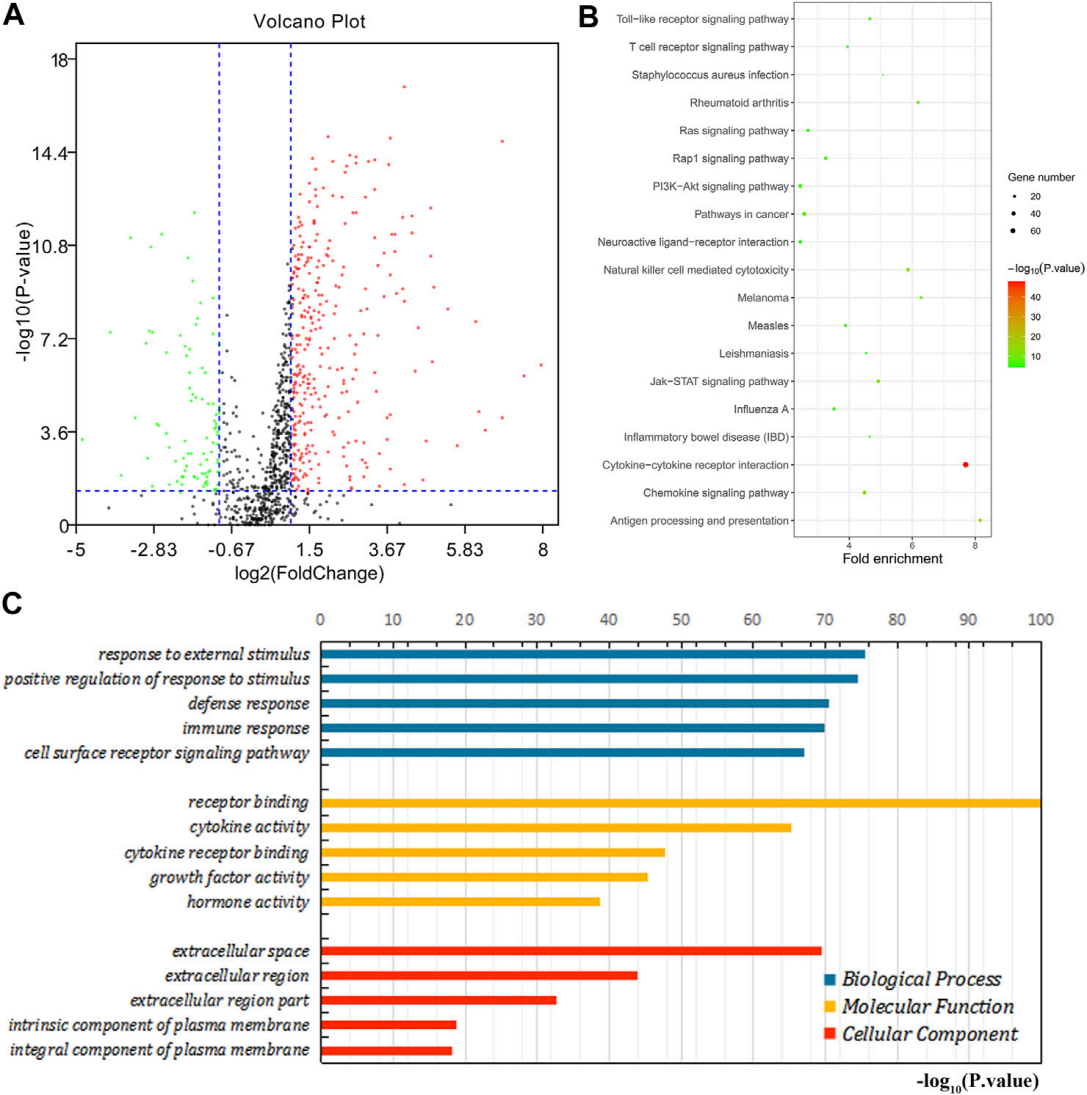
Enrichment analysis of immune-related differential genes. **(A)** The volcano map shows immune-related differential genes. Red is the up-regulated gene and the green is the down-regulated gene. **(B)** KEGG enrichment analysis. **(C)** GO enrichment analysis.

**FIGURE 8:**
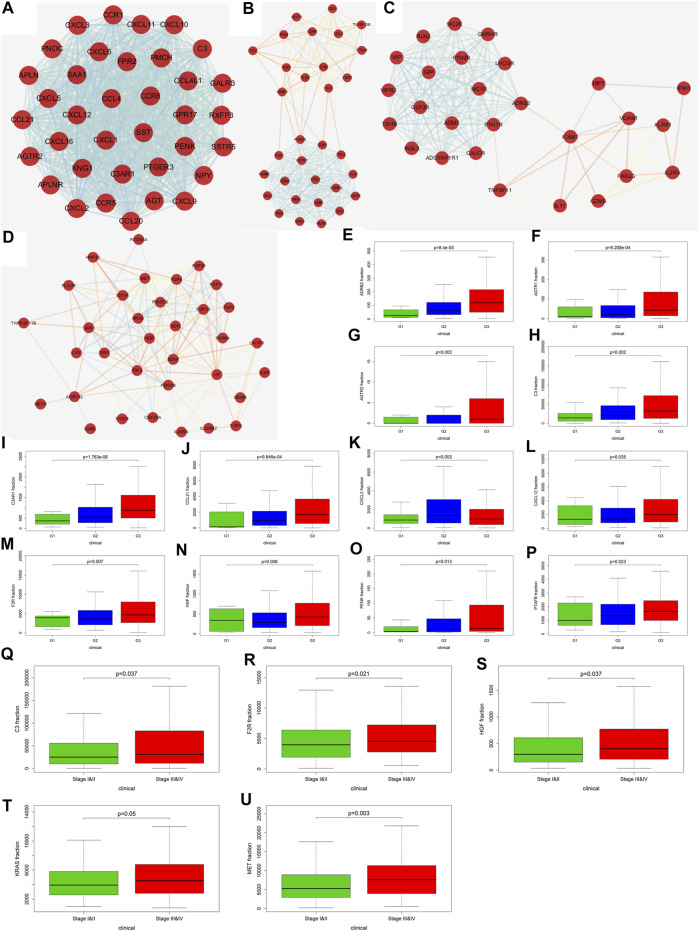
Screening of key genes and correlation analysis with Fuhrman grade and TNM staging. **(A–D)** are the four main modules in the PPI network (Score>10, number of nodes>20). **(E)** (ADRB2), **(F)** (AGTR1), **(G)** (AGTR2), **(H)** (C3), **(I)** (C3AR1), **(J)** (CCL21), **(K)** (CXCL2), **(L)** (CXCL12), **(M)** (F2R), **(N)** (HGF), **(O)** (PENK), and **(P)** (PTAFR) correlation analysis with Fuhrman grade. **(Q)** (C3), **(R)** (F2R), **(S)** (HGF), **(T)** (KRAS), and **(U)** (MET) correlation analysis with TNM staging.

**FIGURE 9:**
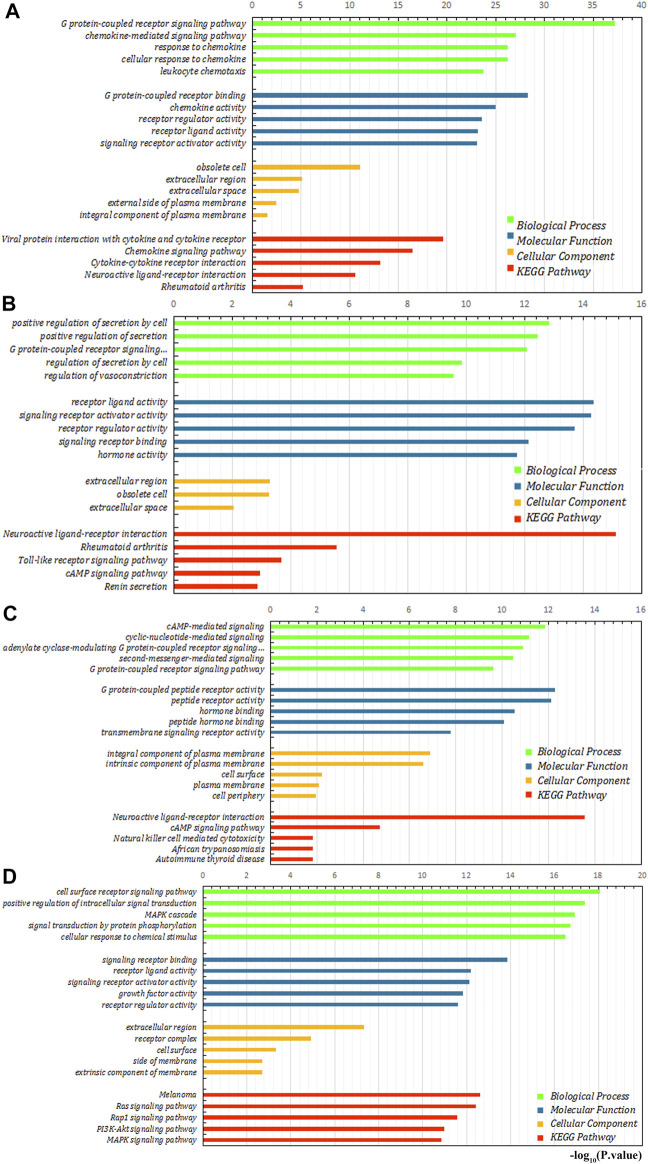
Enrichment analysis of four main modules. **(A–D)** are module **(A)**, module **(B)**, module **(C)**, and module **(D)**, respectively.

We further explored the role of immune infiltration in the progression of GC. First, we performed differential expression analysis in TCGA to obtain differential genes, and used the intersection of differential genes and immune genes to obtain immune-related differential genes. Then, we screened out 411 immune-related differential genes from TCGA, of which 316 were up-regulated genes and 95 were down-regulated genes (Figure 7A). Functional enrichment analysis indicated that these immune-related differential genes were mainly enriched in the response to external stimulus, positive regulation of response to stimulus, receptor binding, cytokine activity (GO terms, Figure 7C), cytokine-cytokine receptor interaction, chemokine signaling pathway and Jak-STAT signaling pathway (KEGG pathways, Figure 7B). In order to explore the interaction between immune-related differential genes, we employed the STRING database and Cytoscape software to construct the PPI network, which included 213 nodes and 1,373 edges. In addition, MCODE analysis was conducted and four modules with at least 20 nodes were identified (Figures 8A–D). Among module A, CXCL3, CXCL5, CCL4L1, CXCL2, AGTR2, CCL21, PENK, CXCL12, C3, and C3AR1 were recognized as important genes for their greater connections with other nodes. In module B, EDN1, PTAFR, AGTR1, and F2R were the hub genes with higher connectivity degree values. Several key immune response genes such as PTH1R, CALCR, and ADRB2 occupy the center of module C. Moreover, KRAS, HGF, and MET were the pivot genes of module D. Then we conducted an enrichment analysis on the four main modules identified. We found that modules were mainly enriched in signal transduction (Figure 9).

### The Relationship Between Immune-Related Genes and GC Progression and Prognosis

**FIGURE 10:**
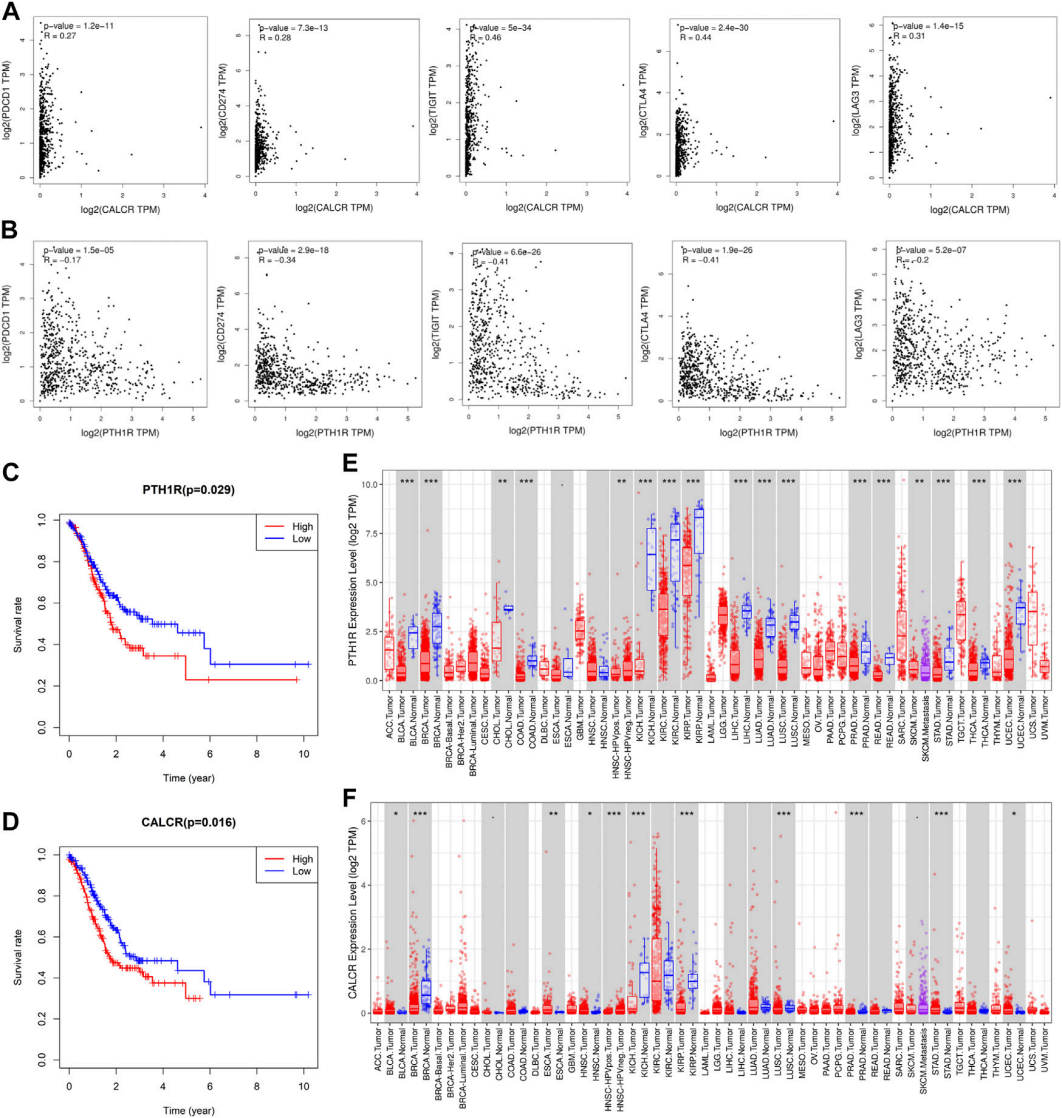
Analysis of the correlation between key genes and immune checkpoints and their prognosis. **(A)** CALCR was significantly positively correlated with the expression of immune checkpoints (PD-1, PD-L1, CTLA4, LAG3, and VSTM3). **(B)** PTH1R is negatively correlated with the expression of immune checkpoints (PD-1, PD-L1, CTLA4, LAG3, and VSTM3). Correlation analysis between **(C)** (PTH1R) and **(D)** (CALCR) and the prognosis of patients with GC. TIMER was used to analyze the expression of **(E)** (PTH1R) and **(F)** (CALCR) in tumors. STAD is gastric adenocarcinoma. Red is tumor tissues and blue is normal tissues. Spearman’s rank test was used to analyze the correlation between immune-related genes and immune checkpoints. Statistical significance: *p < 0.05, **p < 0.01, and ***p < 0.001.

Through the PPI network, we have identified 20 key genes including CXCL3, CXCL5, CCL4L1, CXCL2, AGTR2, CCL21, PENK, CXCL12, C3, C3AR1, EDN1, PTAFR, AGTR1, F2R, PTH1R, CALCR, ADRB2, KRAS, HGF, and MET. Immune checkpoints were one of the reasons for tumor immune escape, we examined the relationship between immune-related genes and major immune checkpoints (PD-1, PD-L1, CTLA4, LAG3, and VSTM3) (Figures 10A,B; Supplementary Figure S3). The results indicate that most immune-related genes were significantly related to immune checkpoint biomarkers. Among them, the relative expression of CALCR, C3, C3AR1, CCL4L1, CCL21, CXCL2, CXCL5, CXCL12, EDN1, F2R, HGF, KRAS, MET, and PTAFR was significantly positively correlated with PD-1, PD-L1, CTLA4, LAG3, and VSTM3 levels, while the relative PTH1R expression was negatively correlated with the levels of PD-1, PD-L1, CTLA4, LAG3, and VSTM3. Then we combined those key genes with Fuhrman grade and TNM staging for analysis. The results showed that ADRB2 (*p* < 0.001), AGTR1 (*p* < 0.001), AGTR2 (*p* = 0.003), C3 (*p* = 0.002), C3AR1 (*p* < 0.001), CCL21 (*p* < 0.001), CXCL2 (*p* = 0.003), CXCL12 (*p* = 0.035), F2R (*p* = 0.007), HGF (*p* = 0.008), PENK (*p* = 0.013), and PTAFR (*p* = 0.023) were closely related to Fuhrman grade, and their expression levels all increased with the increase of Fuhrman grade (Figure 8E–P). C3 (*p* = 0.037), F2R (*p* = 0.021), HGF (*p* = 0.037), KRAS (*p* = 0.05), and MET (*p* = 0.003) were closely related to TNM staging, and their expression levels all increased with the increase of TNM staging (Figures 8Q–U).

Finally, survival analysis of key genes showed that CALCR (*p* = 0.016) and PTH1R (*p* = 0.029) are closely related to the prognosis of GC patients, and both genes are negatively related to patient survival (Figures 10C,D). Then we found that these two genes are expressed in a variety of tumors (Figures 10E,F). In addition, the expression of CALCR in GC tissues was significantly higher than that in normal tissues, while the expression of PTH1R in GC tissues was lower than that in normal tissues. Taken together, these results suggested that CALCR and PTH1R played a critical role in tumor immune escape, which was involved in the carcinogenesis of GC.

## Discussion

Gastric cancer is a common malignant tumor of the digestive tract and one of the malignant tumors with high morbidity and mortality in my country (Ferlay, et al., 2015). The onset of GC is insidious, and the rate of early diagnosis is low. Many patients are at an advanced stage when they see a doctor and have missed the best time for radical surgery (Fu, et al., 2018). In recent years, immunotherapy has developed rapidly, and it has achieved significant results in the treatment of breast cancer (Discovery 2017), lymphoid malignancies (Klausen, et al., 2019), and hematological malignancies (Holmstrom and Hasselbalch 2019). Immunotherapy is a treatment that enhances the function of TIICs in TME to achieve the purpose of eliminating tumors. Therefore, in-depth exploration of the complex interaction between TIICs and tumor cells in TME is beneficial to improve the efficacy of GC immunotherapy. Our research was to screen and identify cells and genes closely related to immune infiltration and clinical outcome in microenvironment of GC, so as to predict and guide immunotherapy. This study not only reveals new cellular and gene targets for GC immunotherapy, but also provides new research ideas for other tumor immunotherapy.

We combined GSE66229 dataset and TCGA to analyze the infiltration of 22 TIICs in TME, and discussed the relationship between TIICs and the clinical characteristics and overall survival of GC patients. We observed that the total T cells in GSE66229 dataset accounted for more than 37%, of which resting CD4^+^ memory T cells accounted for 11.7%, and the total macrophages accounted for about 23.9%, of which M2 macrophages accounted for 10%. In TCGA, the total T cells accounted for more than 42%, of which resting CD4^+^ memory T cells accounted for 16.9%. Total macrophages accounted for about 30%, of which M0 and M2 macrophages both accounted for 11.2%. Both GEO and TCGA data show that resting CD4^+^ memory T cells and M2 macrophages account for a relatively high proportion in TME. Previous studies have also found that these two types of TIICs play an important role in the occurrence and development of tumors (Lewis and Pollard 2006; Sallusto et al., 1999). However, the results of the analysis of the infiltration ratio of M2 macrophages in GC tissues and normal tissues in GEO and TCGA are different. TCGA data shows that the proportion of M2 macrophages infiltrating tumors is higher than that of normal tissues, which is consistent with the results in the literature. And it is different from the results obtained by the analysis of the GSE66229 dataset. This may be because the pathological classification of the cases in the GSE66229 dataset is not all gastric adenocarcinoma, which affects the analysis results of M2 macrophages. Resting CD4^+^ memory T cells maintain immune memory and exert immune protection during tumor metastasis (Ludewig et al., 1999; Wherry et al., 2003), and M2 macrophages can also promote tumor growth and metastasis (Kurahara et al., 2013; Liu and Cao 1992). The in-depth study of resting CD4^+^ memory T cells and M2 macrophages will help to understand the role of TIICs in TME and contribute to the development of GC immunotherapy strategies. Through the correlation analysis between TIICs, we found that CD4^+^ memory T cells may play a key role in the connection between TIICs in GC microenvironment (Shen et al., 2009; Sugasawa et al., 2010).

MSI and TMB have recently attracted widespread attention as promising predictive biomarkers for the efficacy of immunotherapy, and related studies in gastric cancer have gradually increased (Wei et al., 2021; Zhang et al., 2021). Our results show that 13 types of TIICs are significantly related to TMB in the GC microenvironment, and 8 types of TIICs are closely related to MSI. These results strongly suggest that the infiltration of TIICs will affect the response of cancer patients to immune therapy, which will provide new clues to the prognosis of immunotherapy. This study revealed for the first time the relationship between different TIICs and clinical features in GC microenvironment. We found that, as the Fuhrman grade of GC increased, the proportion of CD8^+^ T cells, resting dendritic cells, M1 macrophages and resting mast cells gradually increased, and the proportion of M0 macrophages and activated mast cells gradually decreased. And the lowest proportion of M2 macrophages at moderately differentiated may be caused by too few highly differentiated samples in GC patients. TIICs are significantly related to TNM staging are eosinophils and M2 macrophages, and the proportion of these two kinds of TIICs in the late stage of GC is higher than that in the early stage. There have been many studies on the composition and prognosis of TIICs in TME. A higher proportion of resting NK cells and neutrophils is associated with poor disease-free survival and overall survival (Bense, et al., 2017; Wang, et al., 2018), and the increase in the proportion of mast cell infiltration in tumors is related to the better prognosis of patients (Bo, et al., 2018). This study also made a preliminary exploration of the correlation between immune cell infiltration and survival. The results showed that CD8^+^ T cells and activated CD4^+^ T cells were significantly correlated with improved prognosis, while M2 macrophages indicated a poor prognosis. This is consistent with previous studies in microenvironment of GC (Li L, et al., 2019).

In order to explore the possible mechanism of differences in immune infiltration, this study further analyzed the relative expression of immune genes in TCGA. We obtained immune-related differential genes and enriched them. As expected, most of them are involved in the immune process in TME. Next, we screened out 4 main modules and found that these modules are closely related to the immune process and can activate related pathways. And twenty key genes selected through this module participate in tumor immune escape and promote the recruitment of TIICs in TME, thereby affecting tumor immunity and angiogenesis (Kitago et al., 2006; Mota et al., 2016; Raemdonck et al., 2014; Tian et al., 2018; Vianello et al., 2006; Wang et al., 2017; Yamamoto et al., 2019). These results indicate that there is a complex relationship between TIICs and immune-related genes in TME. This is consistent with previous reports (Deng et al., 2020; Lee et al., 2014; Yang et al., 2020). Studies have shown that the expression of immune checkpoints will significantly affect the effect of immunotherapy (Li X et al., 2019). Most of the 20 key genes we screened are closely related to the expression of immune checkpoints. We also combined analysis of key genes with Fuhrman grade and TNM staging. It is found that twelve genes are closely related to Fuhrman grade, and 5 genes are closely related to TNM staging. In addition, survival analysis found that two genes were significantly related to the prognosis of GC patients. Among them, CALCR is positively correlated with the expression of immune checkpoints, and the expression of PTH1R decreases as the expression of immune checkpoints decreases. Thus, the patients with cancer with high expression of CALCR and low expression of PTH1R may benefit from immunotherapy. TIMER analysis found that CALCR and PTH1R may be promoting and suppressing genes for GC, respectively. Previous studies have failed to explore the relationship between these two genes and immunity, and only a few studies have shown that these genes are related to the occurrence of certain tumors. CALCR is the calcitonin receptor gene. Some studies have shown that the expression of CALCR is increased in neuroendocrine tumors and chronic pancreatitis, and it may inhibit the progression of glioblastoma through the CT-CALCR signal axis (Pal et al., 2018). And some study of CALCR in Glioblastoma shows that it may be suitable as a target for delivering cytotoxic agents (Ostrovskaya et al., 2019). PTH1R is a type 1 receptor for parathyroid hormone. It cooperates with extracellular calcium sensitive receptors (CaSR) to maintain the physiological homeostasis of extracellular calcium ions (Ca2^+^) and lactation. It can promote the proliferation of breast cancer and is closely related to the bone metastasis of breast cancer (Alokail and Peddie 2008; Dittmer et al., 2006; Liang et al., 2012; Yang and Wang 2018). Some studies in GC have shown that PTH1R can be expressed in some gastric cancer cell lines, and reduced PTH1R expression can promote gastric hypergastrinemia and gastric neuroendocrine tumors (Al Menhali et al., 2017; Calvete et al., 2017). Other studies have also shown that PTH1R plays a role in the early-stage progression and proliferation of a variety of tumors (Giovanni et al., 2010; Isabelle et al., 2006; Mitsunobu 2005). However, few studies have explored the role of CALCR and PTH1R in the progression of GC, so in-depth exploration is needed in future experiments.

In summary, this study revealed the infiltration of TIICs and immune-related genes in microenvironment of GC. And they are significantly related to TMB, MSI, clinical progress, and prognosis. Among them, 7 types of TIICs and 12 immune-related genes are closely related to Fuhrman grade, and 2 types of TIICs and 5 immune-related genes are closely related to TNM staging. We also identified 3 TIICs and 2 immune-related genes related to prognosis. These cells and genes can be considered as biomarkers for prognosis, and can also be used as markers for GC immunotherapy. However, the evidence of this study, like other similar bioinformatics studies, is still indirect and requires further experimental verification. Through in-depth research on these TIICs and immune-related genes, we can re-understand the potential relationship between TME and GC immunotherapy and prognosis, and provide a theoretical basis for the development of better immunotherapy strategies.

## Conclusion

There are a clear correlation between TIICs and immune genes and the progression of GC. Changes in the types and numbers of TIICs indicate that immune infiltration plays a complex role in TME, which is closely related to TMB, MSI, and clinical results. TIICs can also predict and influence the prognosis of GC patients. In addition, another important factor affecting the progress and prognosis of GC is immune-related genes. Research on TIICs and immune genes can provide new targets for GC immunotherapy and promote the progress of this field.

## Data Availability

Publicly available datasets were analyzed in this study. This data can be found here: https://portal.gdc.cancer.gov/ https://www.ncbi.nlm.nih.gov/geo/, GSE66229.
