# Supplementary figures and images for: Immune Infiltration in Gastric Cancer Microenvironment and Its Clinical Significance
Source: Front Cell Dev Biol. 2022 Feb 17;9:762029. doi: 10.3389/fcell.2021.762029 (PMC8893596; doi:10.3389/fcell.2021.762029)

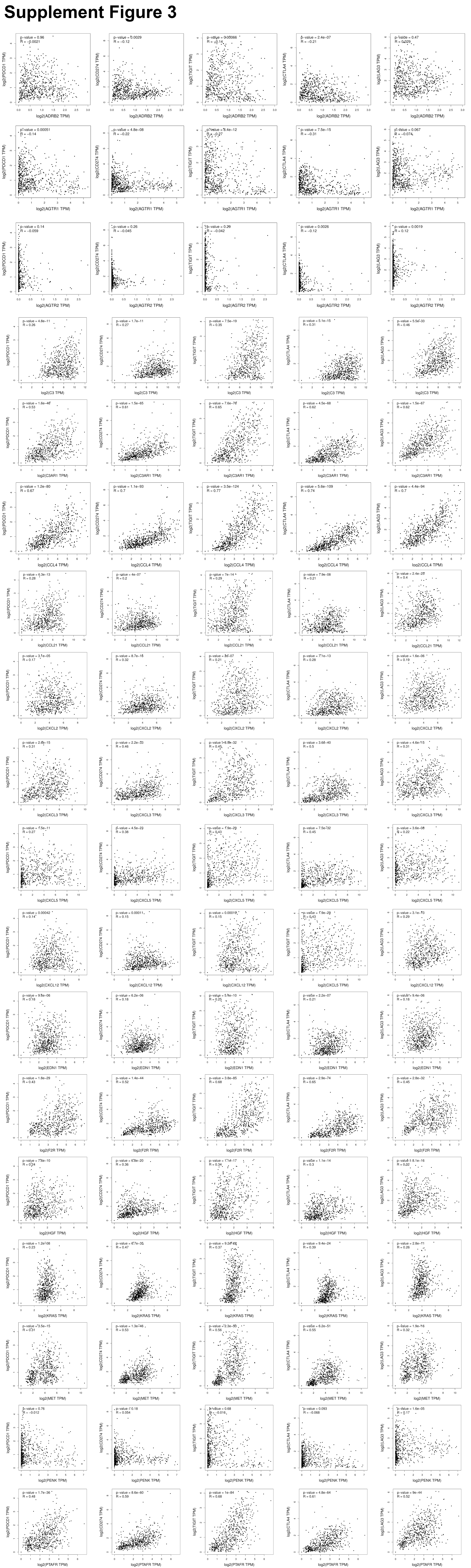

Supplement: Supplementary file 1 [file Image3.TIF]

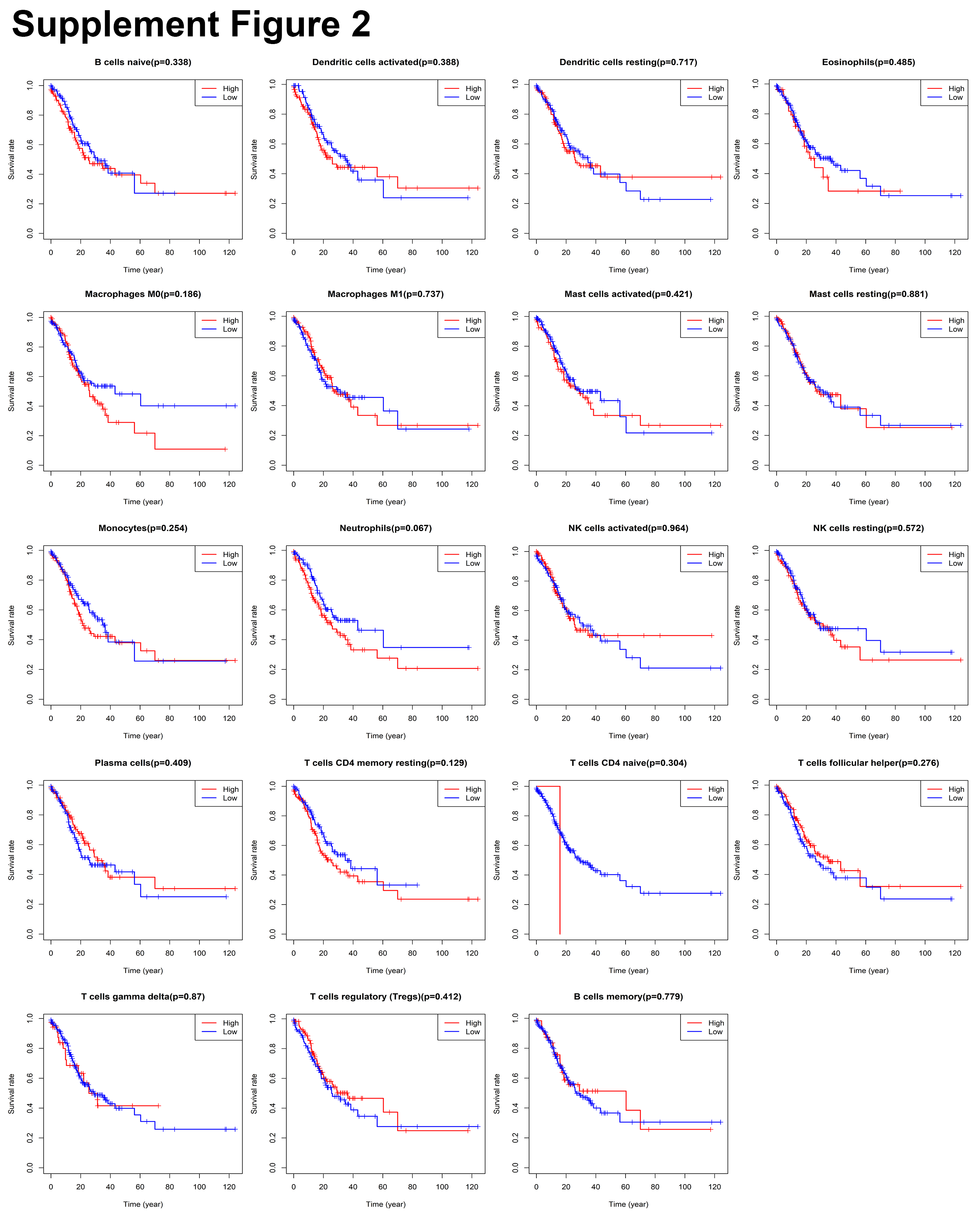

Supplement: Supplementary file 2 [file Image2.TIF]

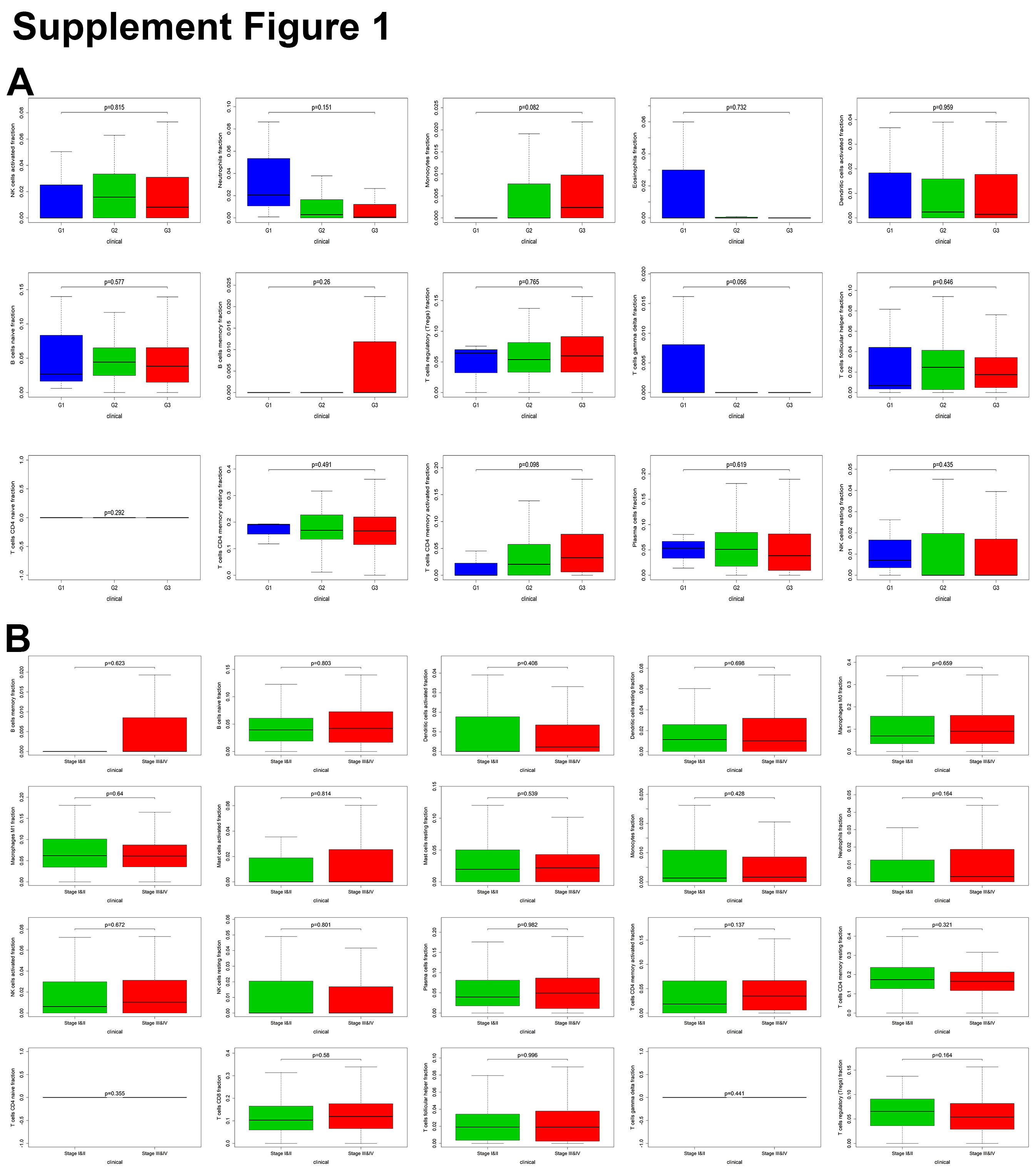

Supplement: Supplementary file 3 [file Image1.TIF]
